# A transparent electrochromic metal-insulator switching device with three-terminal transistor geometry

**DOI:** 10.1038/srep25819

**Published:** 2016-05-13

**Authors:** Takayoshi Katase, Takaki Onozato, Misako Hirono, Taku Mizuno, Hiromichi Ohta

**Affiliations:** 1 Research Institute for Electronic Science, Hokkaido University, N20W10, Kita, Sapporo 001−0020, Japan; 2 Graduate School of Information Science and Technology, Hokkaido University, N14W19, Kita, Sapporo 060−0814, Japan; 3 School of Engineering, Hokkaido University, N13W8, Kita, Sapporo 060−8628, Japan; 4 Graduate School of Engineering, Nagoya University, Furo-cho, Chikusa, Nagoya 464−8603, Japan

## Abstract

Proton and hydroxyl ion play an essential role for tuning functionality of oxides because their electronic state can be controlled by modifying oxygen off-stoichiometry and/or protonation. Tungsten trioxide (WO_3_), a well-known electrochromic (EC) material for smart window, is a wide bandgap insulator, whereas it becomes a metallic conductor H_*x*_WO_3_ by protonation. Although one can utilize electrochromism together with metal-insulator (MI) switching for one device, such EC-MI switching cannot be utilized in current EC devices because of their two-terminal structure with parallel-plate configuration. Here we demonstrate a transparent EC-MI switchable device with three-terminal TFT-type structure using amorphous (a-) WO_3_ channel layer, which was fabricated on glass substrate at room temperature. We used water-infiltrated nano-porous glass, CAN (calcium aluminate with nano-pores), as a liquid-leakage-free solid gate insulator. At virgin state, the device was fully transparent in the visible-light region. For positive gate voltage, the active channel became dark blue, and electrical resistivity of the a-WO_3_ layer drastically decreased with protonation. For negative gate voltage, deprotonation occurred and the active channel returned to transparent insulator. Good cycleability of the present transparent EC-MI switching device would have potential for the development of advanced smart windows.

A transparent electrochromic metal-insulator (EC-MI) switching device that can be electrically switched from a colorless transparent insulator to a colored metallic conductor would be ideal for use in future energy-saving technologies, such as advanced smart-windows. In the OFF state such transparent EC-MI switching device fully transmits visible light, whereas in the ON state it does not transmit light. Furthermore, the device is switched from insulator to conductor at the same time, which can function as an ON/OFF power switch for other electronic devices.

Among many potential electrochromic materials, tungsten trioxide (WO_3_)^1^ shows the greatest suitability for the EC-MI switching devices. Stoichiometric WO_3_ is a transparent insulator with a bandgap (*E*_g_) of 2.6–3.0 eV^2^ and has a defect perovskite-type structure with space group *P*2_1_/*n*, in which A-sites in the ABO_3_ lattices are vacant^3^. If the vacant A-sites become occupied by protons (H^+^), i.e. the formation of tungsten bronze, it becomes an electrical conductor and opaque to visible light following the valence-state change of W ion from W^6+^ to W^5+ ^4. Thus, the protonation/deprotonation of WO_3_ is promising for the realization of simultaneous electrical switching between colorless/colored and insulating/conducting states, as shown in Fig. 1.

Various types of WO_3_-based EC devices have been actively developed for applications in energy-saving smart windows^5^,^6^; however, their MI switching behavior has not been utilized with EC switching simultaneously, because of their two-terminal structure, which involves a parallel-plate electrode configuration. Electrostatic charge modulation using three-terminal thin-film transistors (TFTs)^7^,^8^ on a WO_3_ thin film could be used to realize simultaneous EC-MI switching; however, it remains difficult to fully switch their coloring state, because of limitations associated with the carrier-doping range and modifiable thickness^9^. Several researchers have developed liquid-electrolyte gated transistors^10^,^11^ and successfully demonstrated EC-MI switching of WO_3_. However, because these devices require liquid electrolytes, they are less practical for application, which depends on effective sealing.

Herein, we demonstrate a *liquid-leakage-free* transparent EC-MI switching device. Figure 2a schematically illustrates the device structure, which has a typical three-terminal TFT- geometry composed of an active channel, a gate insulator, and source-drain-gate electrodes. We used an amorphous (a-) WO_3_ thin film as the active channel layer, because EC switching of a-WO_3_ film prepared on glass substrate at room temperature (RT) has been previously reported^12^. The gate insulator consists of an a-12CaO·7Al_2_O_3_ (a-C12A7) thin film with nanoporous structure (calcium aluminate with nanopore, CAN)^13^,^14^. It should be noted that the film porocity of CAN film can be controlled by oxygen pressure (*P*_O2_) during thin-film deposition at RT (porous structure can be observed at *P*_O2 _> 1 Pa), and the nanopores with average diameters of 10−20 nm connect with each other, when the prosity reaches ~30% of fully dense film, leading to the percolation conduction of water in the CAN film. Since C12A7 is a hydroscopic material, water vapor in air is automatically absorbed into the CAN film, like a solid sponge, via the capillary action in the interconnected nanopores. Therefore, water electrolysis can be used in the solid gate insulator. A NiO/ITO (indium tin oxide) bilayer film was used as the gate transparent electrode, and ITO thin films were used as the transparent source and drain electrodes.

The device with leakage-free water can be considered as the nanosized electrochemical cell with a nanogap parallel plate structure, which enables the high electric-field application for ion migration and effective protonation/deprotonation of the a-WO_3_ layer (Fig. 2b). Thus, a gate voltage (*V*_g_) application induces water electrolysis in the CAN film, and produced H^+^ and OH^−^ ions move to protonate the a-WO_3_ layer (WO_3_ + *x*H^+ ^+ *x*e^−^ → H_*x*_WO_3_)^15^ and hydroxylate the NiO layer (NiO + OH^− ^→ NiOOH + e^−^)^16^, respectively. The NiO counter layer is expected to work as the OH^−^ absorber, which maintains a better electrochemical balance and should improve the reversibility and reproducibility of device operation. Alternative positive and negative *V*_g_ applications induce the reversible protonation/deprotonation of a-WO_3_ layer, switching it from a transparent insulator to a dark blue conductor. The present EC-MI switching device with the two combined functions of color changing as a display and electrical switching as a transistor in one device can be reversibly operated at RT without sealing; thus, it may be suitable for a wide-range application in future energy-saving technologies, such as advanced smart-windows.

## Results

### Device fabrication

The EC-MI switching device was fabricated on an alkaline-free glass substrate (Corning^®^ EAGLE XG^®^) by using stencil masks. All the thin-film fabrication processes were conducted at RT using pulsed laser deposition (PLD) with KrF excimer laser (*λ* = 248 nm). Details of the device fabrication process are provided in experimental section. First, 20-nm-thick transparent conducting ITO films (resistivity, *ρ* = 1.0 mΩ cm at RT) were deposited as source and drain electrodes. An 80-nm-thick a-WO_3_ channel film, a 300-nm-thick CAN gate insulator film^13^,^14^, and a NiO (20 nm)/ITO (20 nm) gate electrode film were then deposited in turn. The channel size was 800 μm × 400 μm. The density of a-WO_3_ film was 5.96 g cm^−3^, evaluated by grazing incidence X-ray reflectivity, which corresponds to 82% of ideal density of a WO_3_ crystal (7.29 g cm^−3^)^17^. The AC conductivity of the CAN film was 3.7 × 10^−8 ^S cm^−1^ at RT, slightly less than the 5.6 × 10^−8 ^S cm^−1^ of ultrapure water^18^.

**Figure 2:**
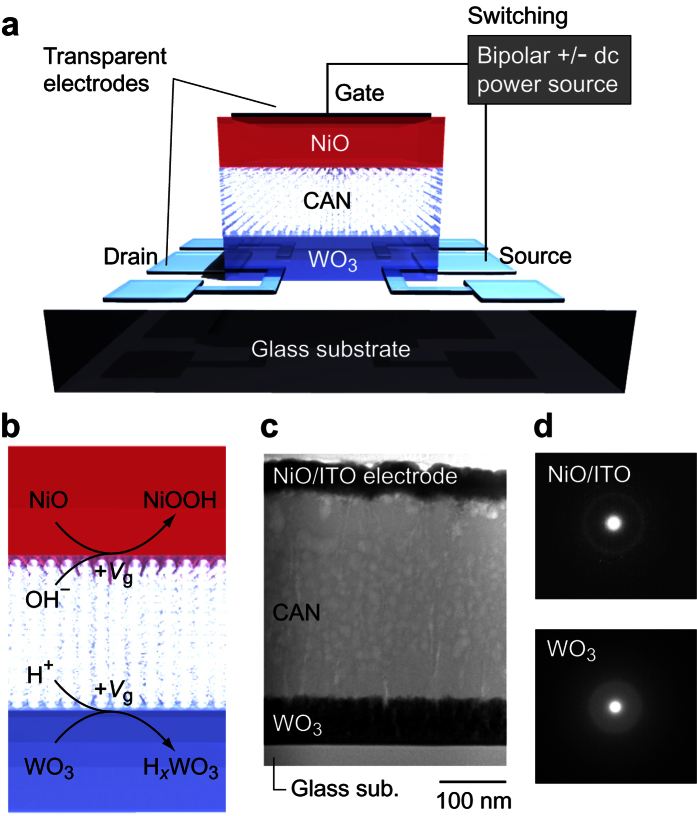
A transparent EC-MI switching device. (**a**) Schematic device structure of three-terminal TFT-type device, composed of a-WO_3_ (80 nm), CAN (300 nm), and NiO (20 nm)/ITO (20 nm) layers. Transparent ITO thin films are used for all the electrodes. (**b**) Device operation mechanism. During the positive *V*_g_ application, protonation of a-WO_3_ layer and hydroxylation of NiO layer occur simultaneously. Conversely, a-H_*x*_WO_3_ and NiOOH return to a-WO_3_ and NiO during the negative *V*_g_ application. (**c**) Cross-sectional BF-STEM image of the device. Trilayer structure is clearly seen. Many light spots in the CAN layer indicate nanopores, which is fully occupied with water. (**d**) Selected area electron diffraction patterns of NiO/ITO layer (upper) and a-WO_3_ layer (lower).

Figure 2c shows a bright-field scanning transmission electron microscopy (BF-STEM, in which heavier atoms appear darker) image of the cross-section of the resultant device, which reveals the multi-layer structure of ITO (20 nm)/NiO (20 nm)/CAN (300 nm)/a-WO_3_ (80 nm) on a glass substrate. Numerous light spots with diameters of 10−20 nm are clearly seen in the CAN film, indicating the presence of high-density nanopores in the CAN film. Figure 2d summarizes the selected-area electron diffraction patterns of the NiO/ITO gate electrode layer (upper) and the a-WO_3_ channel layer (bottom). A broad halo was observed for the a-WO_3_ film, confirming the amorphous structure. Meanwhile, a ring diffraction pattern was seen for NiO/ITO film, originating from polycrystalline nature of NiO film, which was also confirmed by the grazing incidence X-ray diffraction measurements on each thin film.

### Metal-insulator switching

**Figure 3:**
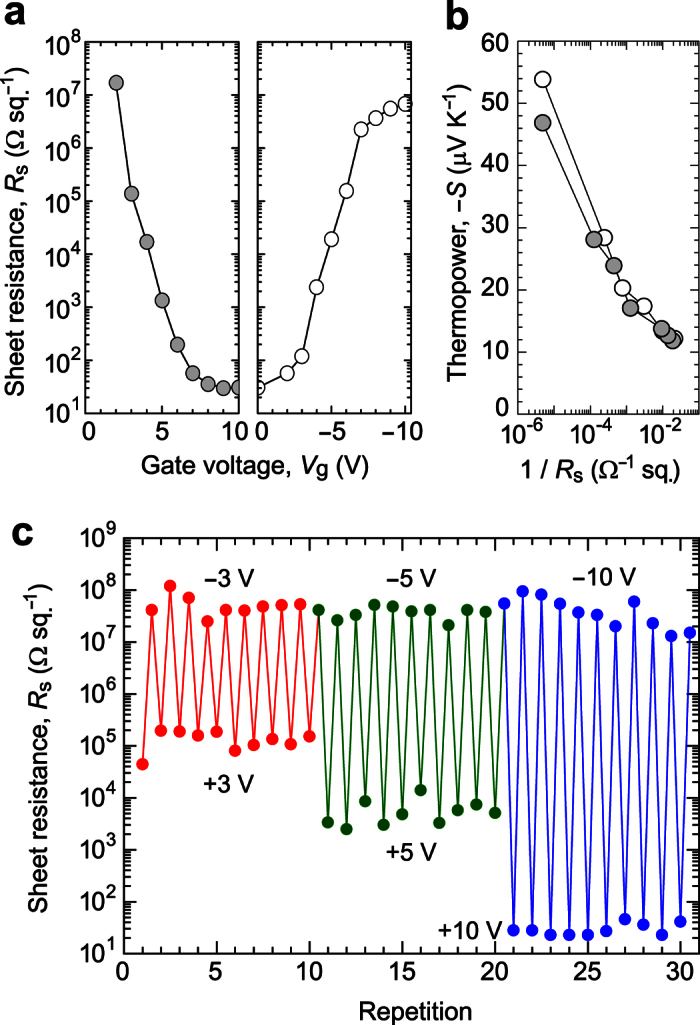
Metal-insulator switching of the transparent EC-MI switching device. (**a**) Sheet resistance (*R*_s_) as a function of applied *V*_g_ for a-WO_3_ layer. *R*_s_ values were measured after *V*_g_ application, where positive *V*_g_ up to +10 V was applied for protonation of a-WO_3_ film (left panel, each *V*_g_ application time of 20 s), and then negative *V*_g_ up to −10 V was applied for deprotonation of a-H_*x*_WO_3_ film (right panel, each *V*_g_ application time of 10 s). (**b**) Thermopower (*S*) as a function of 1/*R*_s_ at RT. The linear relation between –*S* and logarithmic 1/*R*_s_ was reversibly observed by ±*V*_g_ application. (**c**) Repetitive switching property by applying various *V*_g_ = ±3, ±5, and ±10 V. The *R*_s_ modulation ratio can be controlled to be ~10^3^ for ±3 V, ~10^4^ for ±5 V, and ~10^6^ for ±10 V, respectively.

We first evaluated the MI switching of the device by measuring sheet resistance (*R*_s_) and thermopower (*S*) at RT, after applying and subsequently switching off *V*_g_. Figure 3a plots *R*_s_ as a function of applied ±*V*_g_ at RT. First, a positive *V*_g_ up to +10 V was applied to protonate the a-WO_3_ channel (left panel, each *V*_g_ application time of 20 s), and then negative *V*_g_ up to −10 V was applied to deprotonate the a-H_*x*_WO_3_ channel (right panel, each *V*_g_ application time of 10 s). The initial a-WO_3_ channel was highly insulating (*R*_s_ was not in measurable range), but the reduction in *R*_s_ of more than six orders of magnitude was observed by applying positive *V*_g_; the *R*_s_ exponentially decreased from 17 MΩ sq.^−1^ at +2 V and their saturation was observed at ≥+8 V, where the *R*_s_ reached to 30 Ω sq.^−1^ at +10 V. It should be noted that the protonated a-H_*x*_WO_3_ channel was stable under ambient and vacuum conditions at RT after the +*V*_g_ application, confirming the non-volatility of device operation due to the electrochemical reaction. Subsequently, by applying negative *V*_g_ up to −10 V, *R*_s_ clearly recovered, reaching an insulating state (6.8 MΩ sq.^−1^).

We then measured the *S* for a-WO_3_ channel protonated and deprotonated at each ±*V*_g_, because *S* is a good measure to evaluate the electronic-structure change resulting from carrier doping (protonation)^19^,^20^. Figure 3b shows the relationship between *S* and 1/*R*_s_ for the device at RT. *S*-values were always negative, indicating that the channel layer is an n-type conductor. The |*S*| linearly decreased from 47 μV K^−1^ to 11 μV K^−1^ with logarithmic increase in 1/*R*_s_ and the linear relation was reversibly observed with the application of ±*V*_g_, suggesting that protonation of the a-WO_3_ channel provides electrons to the conduction band, and the energy derivative of the electronic density of states (DOS) near the Fermi energy (*E*_F_), 
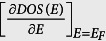
, becomes moderate, resulting in the consequent reduction of |*S*|-values.

Figure 3c shows the results of repeated *R*_s_ switching of the a-WO_3_ channel at various *V*_g_, *e.g*. ±3, ±5, and ±10 V, where the *V*_g_ application time was 20 s for protonation (+*V*_g_) and 10 s for deprotonation (−*V*_g_). Clear cyclability of *R*_s_ switching was observed at each *V*_g_, and the *R*_s_ modulation ratio was largely dependent on the applied *V*_g_; the ON/OFF ratio was ~10^3^ at ±3 V, ~10^4^ at ±5 V, and ~10^6^ at ±10 V. Reversible and reproducible *R*_s_ switching with large ON/OFF ratios occurred rapidly on the second-time scale.

The device operation is considered to be largely dependent on the time-scaled processes consisted of ionic polarization in water, electric double layer formation, electrochemical reaction, and H^+^ diffusion in a-WO_3_ channel under the *V*_g_ applications. The rate-determining step among them for the protonation of WO_3_ has been reported to be the surface reaction process^21^,^22^. Therefore, the difference of the application time of +*V*_g_ (protonation) and −*V*_g_ (deprotonation) should originate from the activation barrier for the surface reaction, i.e. the in-diffusion and out-diffusion of H^+^ transport have different interfacial resistances^23^, which can be seen in the different gate current flowing in the device even at the same ±*V*_g_ (it will be shown later). In addition, the *V*_g_-dependent ON/OFF ratio of the *R*_s_ modulation (Fig. 3c) suggests that the H^+^ diffuses from the surface and the penetration depth along the out-of-plane direction can be controlled by the *V*_g_, where the entire channel region is protonated by applying *V*_g_ ≥ 8 V.

### Electrochromic switching

**Figure 4:**
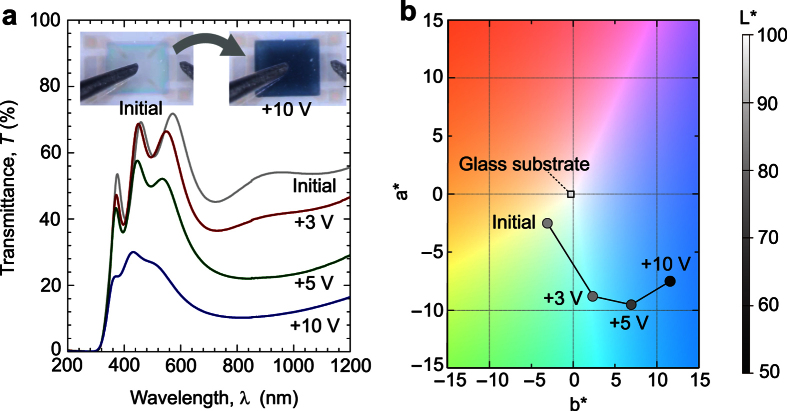
Electrochromic switching of the transparent EC-MI switching device. (**a**) Optical transmission spectra of the device. Significant decrease of the transmission is seen with increasing *V*_g_. The inset shows the photographs of the device; after the application of *V*_g_ = +10 V, the device became opaque/dark blue (right), whereas the device was colorless transparent at the initial state (left). (**b**) L^*^a^*^b^*^ color space plot of the device at each state in (**a**).

We next evaluated the EC switching of the device. Figure 4a shows the optical transmission spectra of the device in the initial state (black line) and protonated states at +3 V (red line), +5 V (green line), and +10 V (blue line). The transmission (*T*) of the device was largely changed by the applied +*V*_g_. The initial device was, to some extent, transparent in the visible light region. After protonation, *T* at *λ* = 700 nm reduced to 24% that of the initial state (transmission modulation of 35%). The inset shows the device picture at the initial and protonated states (+10 V); the color clearly changes from transparent colorless to opaque dark blue, as can be seen in L^*^a^*^b^*^ color space (Fig. 4b), reflecting the optical modulation of the a-WO_3_ channel by RT protonation. Thus, simultaneous EC-MI switching was realized in the device. It should be noted that there is no direct evidence for the hydroxylation of NiO to make NiOOH in this device, which should also affect the optical transmission spectra; NiO changes its color from transparent to deep brown by hydroxylation^24^. However, considering that the no gas generation was confirmed in this device, which is indirect evidence to suggest the OH^−^ ion absorption in NiO counter layer, and there are many reports on the hydroxylation of NiO with aqueous solutions and their effect on electrochromic devices^24^, NiO should play as OH^−^ absorption layer but further confirmation is necessary.

## Discussion

**Figure 5:**
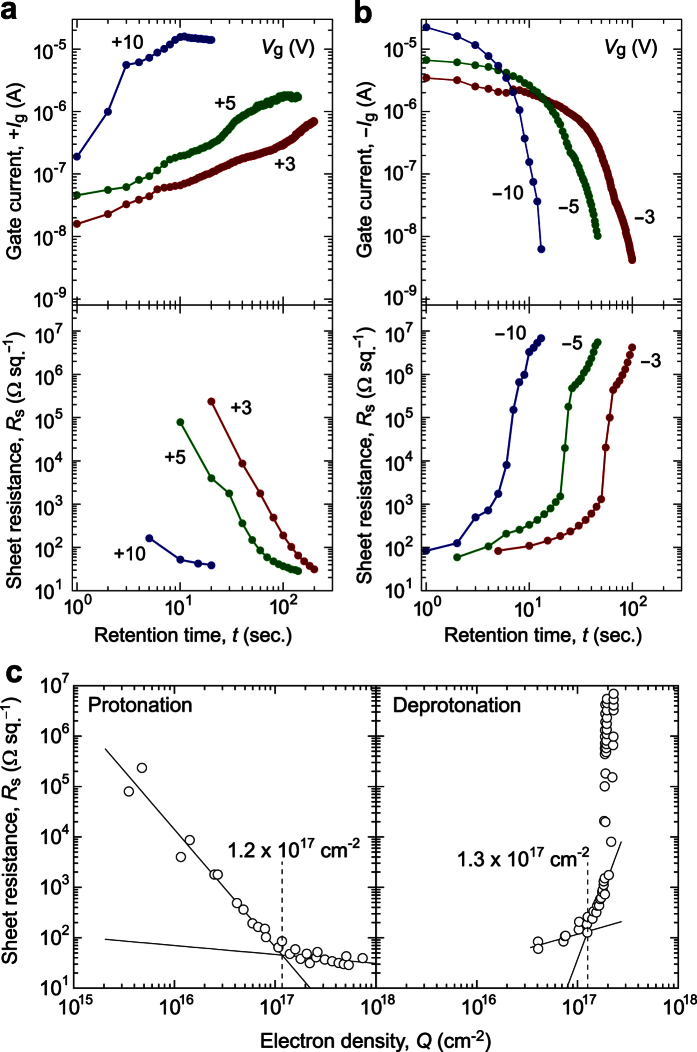
Switching mechanism of the transparent EC-MI switching device. (**a,b**) Retention time (*t*) dependence of gate current (*I*_g_, upper) and sheet resistance (*R*_s_, lower) under application of various ±*V*_g_’s from 3, 5, and 10 V, where (**a**) +*V*_g_ was initially applied for protonation and (**b**) −*V*_g_ was subsequently applied for deprotonation. (**c**) Electron-density (*Q*) dependence of *R*_s_ under application of the various V_g_’s. The *Q* was calculated as the integrated value of the *I*_g_–*t* plots in (**a,b**). The universal changes in *R*_s_ are presented by the black lines. The critical *Q* of protonation and deprotonation was 1.2–1.3 × 10^17 ^cm^−2^, which corresponds well with the ideal *Q* (1.3 × 10^17 ^cm^−2^) required for the 100% protonation/deprotonation reaction of WO_3_ + H^+^ + e^−^ 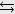 HWO_3_.

To clarify the device operation mechanism, we investigated the relationship between the flowing current and *R*_s_ of the a-WO_3_ channel under various *V*_g_ conditions. Figure 5a,b summarize the retention-time (*t*) dependence of the gate current (*I*_g_) along with *R*_s_ for the a-WO_3_ channel under ±*V*_g_ values of 3, 5, and 10 V, where the +*V*_g_ was initially applied for protonation (Fig. 5a) and −*V*_g_ was subsequently applied for deprotonation (Fig. 5b). *I*_g_ and *R*_s_ were measured during and after the *V*_g_ application, respectively. Upon +*V*_g_ application (Fig. 5a), +*I*_g_ increased with *t* for all applied +*V*_g_ (the *I*_g_ at +10 V showed an irregular jump and exponentially increased and then exhibited the saturation at last), while *R*_s_ simultaneously decreased, indicating that electrochemical protonation of the a-WO_3_ channel occurred. Meanwhile, upon −*V*_g_ application (Fig. 5b), −*I*_g_ decreased with *t* at each −*V*_g_ while *R*_s_ simultaneously increased due to electrochemical deprotonation. This observation suggests the following device operation mechanism. The +*V*_g_ application first accumulates charge carriers at film surface via electrostatic field effect with small +*I*_g_, resulting in the formation of parallel-plate capacitor, i.e. the electric field starts to be well applied on the channel surface, and a further +*V*_g_ application generates dissociated H^+^ and OH^−^ ions and attracted them to each film surface, which should be primary origin of the significant increase of *I*_g_ at the initial stage. The H^+^ and OH^−^ ions diffuse from the surface to bulk region of a-WO_3_ film and NiO counter layer, where the improvement of conductivity of each layer exponentially increases the *I*_g_.

We then compared the variation in *R*_s_ of the a-WO_3_ channel with respect to applied electron density (Fig. 5c) estimated as 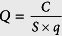, where *C* is total coulomb amount calculated from the integral value of the *I*_g_–*t* plots in Fig. 5a,b, *S* is the surface area of a-WO_3_ channel, and *q* is elementary charge, respectively. For protonation (+*V*_g_ application), *R*_s_ steeply decreased with increasing *Q* up to 1.2 × 10^17 ^cm^−2^ (≡1.5 × 10^22 ^cm^−3^) and then kept unchanged with further electron injection. In contrast, for deprotonation (−*V*_g_ application), *R*_s_ increased moderately at *Q* up to 1.3 × 10^17 ^cm^−2^ (≡1.6 × 10^22 ^cm^−3^), after which it sharply switched to insulating state. The *Q* described here does not mean the carrier concentration in the film but electrochemically-used electron density (≈H^+^ concentration). Referring to the previous report on H^+^ implantation^25^, almost the same dose density (~10^17^ cm^−2^) of H^+^ was used for metallization of WO_3_, but the carrier concentration was measured to be 7.2 × 10^20 ^cm^−3^, where the efficiency of carrier generation was only ~60%; this situation should be equivalent for the present result. The change of *R*_s_ with *Q* seems to occur differently by protonation and deprotonation processes, which should come from the inhomogeneous H^+^ distribution in a-H_*x*_WO_3_ channel along the out-of-plane direction and the H^+^-concentration dependent activity (chemical potential) of H^+^ transport^26^. The critical *Q* of MI switching of the a-WO_3_ channel corresponds with the ideal *Q* value (1.3 × 10^17^ cm^−2^) required for the 100% protonation/deprotonation of a-WO_3_, according to the following reaction: WO_3_ + H^+^ + e^−^ 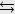 HWO_3_ (the *Q* is estimated by 
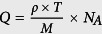
, where *M* is molar mass of WO_3_, *ρ* is the film density, *T* is the film thickness, and *N*_A_ is Avogadro constant, respectively).

The activation energy (*E*_a_) of the electrical conductivity for the fully protonated a-H_*x*_WO_3_ channel that was determined by *R*_s_–*T* measurements (Fig. S1) was *E*_a_ = 4.3 × 10^−3 ^eV. This was an order of magnitude smaller than *E*_a_ = 5.0 × 10^−2 ^eV reported for the a-H_*x*_WO_3_ film (*x* = 0.32)^27^. Since the *E*_a_ is a function of *x* due to the band filling, the much lower *E*_a_ in the a-WO_3_ channel layer supports that it is effectively protonated by water-electrolysis with high electric-field application in the present TFT-type structure. This result also supports the above conclusion. In addition, *R*_s_ decreased and increased along the universal line under all values of *V*_g_ (Fig. 5c), indicating that all the provided electrons were used for electrochemical protonation/deprotonation of the a-WO_3_ channel, obeying Faraday’s laws of electrolysis, and that the device operation can be controlled by the current density.

The present device with leakage-free water can be reversibly switched from a colorless transparent insulator to a colored metallic conductor in a short amount of time (~10 s, see Video S1). In the ON state, visible light cannot transmit through the device, whereas it can fully transmit in the OFF state. The device can also function as an ON/OFF power switch for other electronic devices. The device is mainly composed of amorphous oxide films, which can be deposited at RT with no substrate heating required. This means there are no limitations on the type of substrate materials that can be used for the device. Moreover, the device can be operated without sealing thanks to the liquid-leakage-free CAN gate insulator. These features are suitable for the development of large-area devices and mass production; thus, the present device may find practical application in future energy-saving technologies, such as advanced smart windows.

In summary, we have demonstrated a liquid-leakage-free transparent EC-MI switching device, which has a three-terminal TFT-type structure consisting of transparent oxide thin films of a-WO_3_ active channel, CAN gate insulator, NiO/ITO gate electrode, and ITO source-drain electrodes. At initial state, the device was fully transparent in the visible light region and the WO_3_ channel was insulator. For +*V*_g_ application, the device became dark-blue-colored state and the *R*_s_ of the WO_3_ channel drastically decreased due to the protonation of a-WO_3_ channel. For −*V*_g_ application, deprotonation of a-WO_3_ channel occurred and the device returned to colorless-transparent-insulator state. The reversible EC operation (transmission modulation of 35% at *λ* of 700 nm) and MI switching (*R*_s_-modulation ratio ~10^6^) were simultaneously demonstrated. The present transparent EC-MI switching device with leakage-free water is composed mostly of amorphous oxide thin films, which can be deposited at RT, and can be operated without sealing. Such low cost device will find the practical application for future energy saving technologies such as advanced smart-windows.

## Methods

### Device fabrication

The present liquid-leakage-free transparent EC-MI switching device (active channel area: 400 μm in width and 800 μm in length) was fabricated on an alkaline-free glass substrate (Corning^®^ EAGLE XG^®^, substrate size: 10 × 10 × 0.7 mm^3^) by pulsed laser deposition (PLD) using stencil masks. All the thin-film fabrication was conducted at RT, where a KrF excimer laser (wavelength of 248 nm, repetition rate of 10 Hz) was used to ablate ceramic target disks. First, 20-nm-thick metallic ITO films were deposited at O_2_ pressure (*P*_O2_) of 4 Pa as the transparent source and drain electrodes. Then, a-WO_3_ channel layer was deposited under *P*_O2_ of 7 Pa, where the deposition rate of a-WO_3_ film was 6 nm min^−1^. 300-nm-thick CAN gate insulator was deposited under *P*_O2_ of 5 Pa to make CAN film nanoporous structure^13^,^14^. NiO (20 nm)/ITO (20 nm) bilayer film was deposited as the transparent gate electrode on the CAN film surface, where the nanopores in the CAN film is small enough to prevent the NiO/ITO film penetrate into the CAN film and reach a-WO_3_ layer during the deposition.

### Structural characterization

Crystallinity of the fabricated thin films were investigated by grazing incidence X-ray diffraction analyses (Cu Kα_1_, ATX-G, Rigaku Co.), which revealed that all oxide layers were amorphous in nature expect for NiO polycrystalline film. Cross-sectional thin-film samples for TEM observations were prepared by focused-ion-beam (FIB) micro-sampling technique, in which the multilayer structure region of the TFTs was cutout and thinned by FIB (FB-2000A, HITACHI) to obtain samples for cross-sectional observation. The cross-sectional microstructure and electron diffraction pattern of the a-WO_3_ devices were examined by high-resolution TEM and STEM (JEM-ARM200F, 200 kV, JEOL Ltd.).

### Electrical and optical property measurents

*I*_g_ was measured between the gate and source electrodes during the *V*_g_ application using a source measurement unit (Keithley 2450). The electrical and optical properties were measured after switching the *V*_g_ off, because of the non-volatile device operation due to the electrochemical reaction. *R*_s_ were measured by the d.c. four-point probe method (van der Pauw configuration). For the retention time dependence of *I*_g_ and *R*_s_ (Fig. 5a,b), *I*_g_ was measured at 1 s intervals, and *R*_s_ was measured at each interval of 20 s for +3 V, 10 s for +5 V, 5 s for +10 V, 5 s for −3 V, 2 s for −5 V, 1 s for −10 V, respectively. Thermopower (*S*) was measured by giving a temperature difference (Δ*T*) of ~4 K in the film using two Peltier devices, where the actual temperatures of both sides of a-WO_3_ channel layer were monitored by two tiny thermocouples. The thermo-electromotive force (Δ*V*) and Δ*T* were simultaneously measured, and the *S*-values were obtained from the linear slope of the Δ*V*–Δ*T* plots. Optical transmission spectra were measured by UV-Vis/NIR microscope with the light irradiation area of 100 μm in diameter (MSV-5200, JASCO). The relative humidity value, at which the device operation was tested, was ~30% at 25 °C. Since the present device electrochemically operates in a cycled process between a-WO_3_ cathodic layer (WO_3_ + *x*H^+^ + *x*e^−^ → H_*x*_WO_3_) and NiO anodic layer (NiO + OH^−^ → NiOOH + e^−^), the water in CAN gate insulator should not be lost during the device operation and the CAN does not degrade after many cycles because of the no gas generation. However, it is considered that the humidity in air slightly affect the device operation, which should be tested in the near future.

## Additional Information

**How to cite this article**: Katase, T. *et al*. A transparent electrochromic metal-insulator switching device with three-terminal transistor geometry. *Sci. Rep*. **6**, 25819; doi: 10.1038/srep25819 (2016).

## Supplementary Material

Supplementary Information

Supplementary Video S1

## Figures and Tables

**Figure 1:**
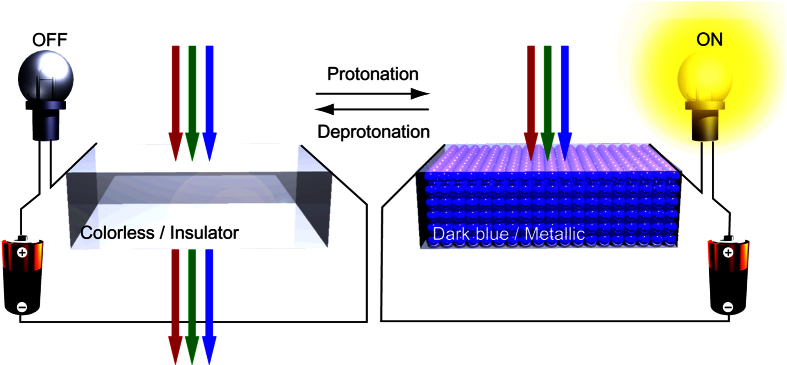
Concept of an electrochromic metal-insulator switching device. The device can be switched from a colorless transparent/insulator state to a dark blue/metallic state simultaneously by electrochemical protonation/deprotonation at RT in air. In the ON state, the visible light cannot be transmitted through the device, whereas it can be fully transmitted in the OFF state. Further, the device can function as an ON/OFF power switch for other electronic devices at the same time. Such a device would be useful for advanced smart window application.
