# Supplementary material for: A transparent electrochromic metal-insulator switching device with three-terminal transistor geometry
Source: Sci Rep. 2016 May 13;6:25819. doi: 10.1038/srep25819 (PMC4865842; doi:10.1038/srep25819)
Supplement: Supplementary Information [file srep25819-s1.pdf]

Supplementary Information Fig. S1 and Video S1

A transparent electrochromic metal-insulator switching device with three-terminal transistor geometry

Takayoshi Katase[1], Takaki Onozato[2], Misako Hirono[3], Taku Mizuno[4], and Hiromichi Ohta[1]

[1]Research Institute for Electronic Science, Hokkaido University, N20W10, Kita, Sapporo 001−0020, Japan
[2] Graduate School of Information Science and Technology, Hokkaido University, N14W19, Kita, Sapporo 060−0814, Japan
[3] School of Engineering, Hokkaido University, N13W8, Kita, Sapporo 060−8628, Japan
[4] Graduate School of Engineering, Nagoya University, Furo-cho, Chikusa, Nagoya 464−8603, Japan

Correspondence and requests for materials should be addressed to
T.K. (katase@es.hokudai.ac.jp) and H.O. (hiromichi.ohta@es.hokudai.ac.jp)

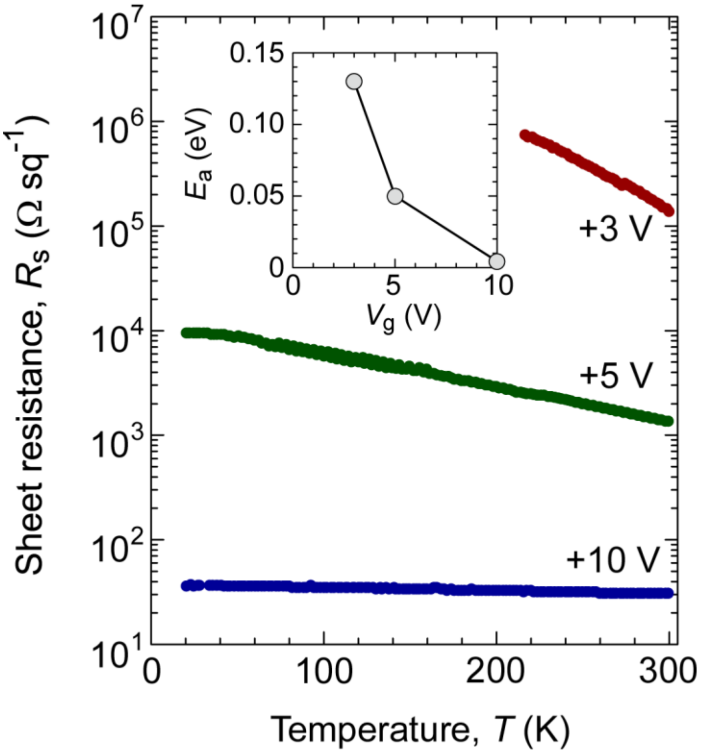

Fig. S1. Temperature dependence of sheet resistance (R_s) for a-WO_3 channel layer of the device protonated at V_g = +3 V, +5 V, and +10 V applied for 20 s. All the R_s–T curves showed semiconducting behavior; the exponential increase of R_s was observed with respect to temperature, which is consistent with the reports that electrical conductivity in a-WO_3,[1] a-H_xWO_3,[2] and a-Na_xWO_3 films[3] follows the variable range hopping model between localized electronic states. The inset shows the activation energy (E_a), estimated from ln (1/R_s) vs. 1000/T relation at 300–200 K range. The E_a largely decreased from 1.3×10^{-1} eV to 4.3×10^{-3} eV, and the R_s–T curve at +10 V showed almost no temperature dependence. Considering that the minimum E_a of electrochemically prepared a-H_xWO_3 film (x = 0.32) was reported to be 5.0×10^{-2} eV,[2] the proton concentration is much higher in the present a-H_xWO_3 channel layer.

Video S1. By applying the V_g = ±10 V for a-WO_3 device at RT in air, colorless transparent insulator was reversibly switched to colored metallic conductor within 10 s.

Reference

[1] Bechinger, C., Herminghaus, S. & Leiderer, P. Photoinduced doping of thin amorphous WO_3 films. *Thin Solid Films* **239**, 156 (1994).

[2] Crandall, R. S. & Faughnan, B. W. Electronic transport in amorphous H_xWO_3. *Phys. Rev. Lett.* **39**, 232 (1977).

[3] Lekshmi, I. C., Gayen, A., Prasad, V., Subramanyam, S. V. & Hegde, M. S. Structure and electrical properties of sodium tungsten bronzes thin films. *Mater. Res. Bull.* **37**, 1815 (2002).
